# *Bombus terrestris* in a mass‐flowering pollinator‐dependent crop: A mutualistic relationship?

**DOI:** 10.1002/ece3.4784

**Published:** 2018-12-18

**Authors:** Jessica L. Knapp, Matthias A. Becher, Charlotte C. Rankin, Grace Twiston‐Davies, Juliet L. Osborne

**Affiliations:** ^1^ University of Exeter Penryn Cornwall UK

**Keywords:** *Bombus terrestris*, *Cucurbita*, mass‐flowering crop, pollen diet, pollination, pollinator populations

## Abstract

Bumblebees (*Bombus* spp.) rely on an abundant and diverse selection of floral resources to meet their nutritional requirements. In farmed landscapes, mass‐flowering crops can provide an important forage resource for bumblebees, with increased visitation from bumblebees into mass‐flowering crops having an additional benefit to growers who require pollination services. This study explores the mutualistic relationship between *Bombus terrestris* L. (buff‐tailed bumblebee), a common species in European farmland, and the mass‐flowering crop courgette (*Cucurbita pepo *L.) to see how effective *B. terrestris *is at pollinating courgette and in return how courgette may affect *B. terrestris *colony dynamics. By combining empirical data on nectar and pollen availability with model simulations using the novel bumblebee model *Bumble*‐BEEHAVE, we were able to quantify and simulate for the first time, the importance of courgette as a mass‐flowering forage resource for bumblebees. Courgette provides vast quantities of nectar to ensure a high visitation rate, which combined with abundant pollen grains, enables *B. terrestris *to have a high pollination potential. While *B. terrestris* showed a strong fidelity to courgette flowers for nectar, courgette pollen was not found in any pollen loads from returning foragers. Nonetheless, model simulations showed that early season courgette (nectar) increased the number of hibernating queens, colonies, and adult workers in the modeled landscapes. *Synthesis and applications*. Courgette has the potential to improve bumblebee population dynamics; however, the lack of evidence of the bees collecting courgette pollen in this study suggests that bees can only benefit from this transient nectar source if alternative floral resources, particularly pollen, are also available to fulfill bees’ nutritional requirements in space and time. Therefore, providing additional forage resources could simultaneously improve pollination services and bumblebee populations.

## 1 INTRODUCTION

Loss of floral resources due to changes in land management is generally thought to be the primary driver of reported declines in pollinator populations Brown and Paxton (2009). This is because generalist flower visitors such as bumblebees (*Bombus* spp.) rely on an abundant and diverse selection of floral resources for nectar and pollen to meet their energy requirements: nectar is rich in sugars, a source of energy, and pollen is rich in protein which is essential for growth and development (Rotheray, Osborne, & Goulson, 2017).

In farmland, mass‐flowering crops are often the intended forage resource because insect visitation can result in pollination, and therefore, increased yield (Pufal, Steffan‐dewenter, & Klein, 2017). This is the case for courgette (*Cucurbita pepo *L.) where pollination, particularly by bumblebee species has been shown to increase yield by 39% (Knapp & Osborne, 2017). Indeed *Bombus impatiens *C. (a North American species) has been observed to be a highly effective pollinator in *Cucurbita* crops, depositing more than three times the number of pollen grains per stigma compared to *Apis mellifera* L. and *Peponapis pruinosa* S. (Artz & Nault, 2011). Quantifying the effectiveness of individual pollinator species can help growers target their pollination management to species most likely to increase yields (Ne'eman, Jürgens, Newstrom‐Lloyd, Potts, & Dafni, 2010).

While mass‐flowering crops may enhance pollinator densities (Westphal, Steffan‐Dewenter, & Tscharntke, 2003), it is largely unknown if this is due to a transient movement of bees between patches of forage or due to an actual increase in colony growth (Holzschuh et al., 2016). This is because mass‐flowering crops only provide temporary pulses of nectar and pollen unlike natural areas, with higher floral species richness, which are able to provide resources that are more stable over time (Montero‐Castaño, Ortiz‐Sánchez, & Vilà, 2016). Nonetheless, intense flowering periods and large areas of mass‐flowering crops in the landscape may still benefit pollinators spatially and temporally, potentially improving pollination and boosting bee populations.

Since accurately studying bumblebee colony development in a field setting can be difficult (Westphal, Steffan‐Dewenter, & Tscharntke, 2009; Wood, Holland, Hughes, & Goulson, 2015), this study uses an in‐silico approach to simulate the population dynamics of *Bombus terrestris* L. in landscapes with and without courgette fields using the agent‐based model *Bumble*‐BEEHAVE (Becher et al., 2018). Although other bumblebee models exist (Crone & Williams, 2016; Häussler, Sahlin, Baey, Smith, & Clough, 2017; Olsson, Bolin, Smith, & Lonsdorf, 2015), *Bumble*‐BEEHAVE is uniquely able to simulate the effects of multifactorial stressors on bumblebee survival at individual, colony and population levels on a daily basis, based on nectar and pollen sources which are approximated from real landscape maps of study sites.

This study explores the mutualistic relationship between *B. terrestris*, a common bumblebee visitor to courgette fields in the United Kingdom (Knapp & Osborne, 2017), and the mass‐flowering crop courgette to ask: (a) How much pollen and nectar do courgette crops provide? (b) Is *B. terrestris* an effective pollinator (in terms of visitation rate and pollen transfer) of courgette? and (c) How does courgette affect *B. terrestris* colony development at a landscape scale (using *Bumble*‐BEEHAVE)?

**Figure 1:**
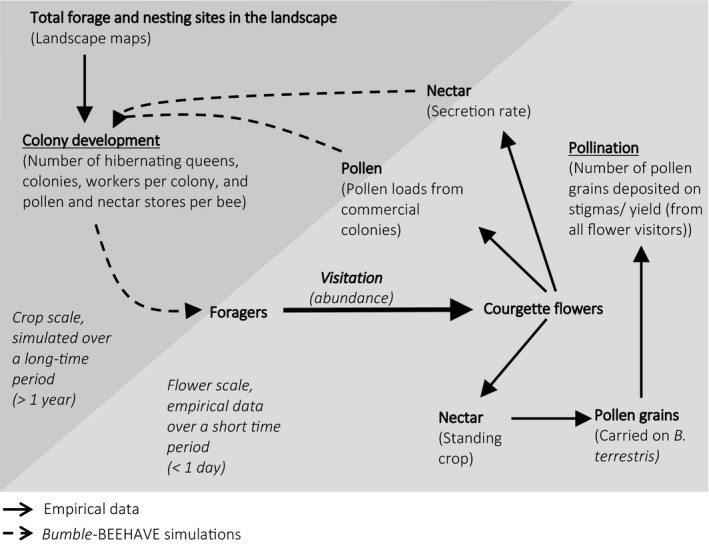
Concept explored in this study of the mutualistic relationship between *Bombus terrestris* and courgette. Solid arrows show where empirical data were collected, and dashed arrows show where results were created from *Bumble*‐BEEHAVE simulations (using BEE‐STEWARD software). Methods for each stage are in parenthesis

To answer these questions, we quantified the potential pollination efficiency of *B. terrestris* in courgette as well as the extent to which courgette fulfills bees’ requirements for pollen and nectar (Figure 1). Combining empirical data with model simulations allowed for the relationship between courgette and *B. terrestris* to be explored at different spatial (flower/crop) and temporal (day/year) scales (Figure 1).

## 2 MATERIALS AND METHODS

### 2.1 Study species

Courgette is monoecious with predominate staminate flowers until pistillate flowers gradually dominate over a season. Within a single day, both types of flower start opening around 05:30 hours before closing around 12:00 hours on the same day, and they do not open again. Flower anthesis is not thought to be directly affected by climatic events such as rainfall (Nepi & Pacini, 1993).

In the United Kingdom, courgette is usually grown over two cropping periods with flowering and harvesting lasting around 5 weeks at two separate sites, often several kilometers apart, to ensure a constant supply of courgette from the beginning of June until the end of August. Hereafter, the first cropping period is referred to as “early courgette” and the second cropping period is referred to as “late courgette.”.

Although all bee species visiting courgette were recorded during pollinator surveys, *B. terrestris* was the focus of this study because of their natural abundance at study sites and availability as commercial colonies (Biobest Biological Systems, Belgium) which were required to quantify the proportion of courgette pollen in *B. terrestris’* diet (Figure 1). Colonies were placed in each field, with sugar water but no additional pollen at a density of three colonies per field.

### 2.2 Study sites

The empirical data for this study (Figure 1) were collected in 19 courgettes (var. “Tosca”) fields in Cornwall, UK from the beginning of June until the end of August in 2016 (five fields) and 2017 (14 fields). Each field had an average field size of 3.6 ± 0.3 ha *SE* and was situated at least 2 km from any other courgette field so that pollinator communities were unlikely to be shared between fields (Vaissière, Freitas, & Gemmill‐Herren, 2011).

All courgettes were grown conventionally in outdoor (as opposed to protected) conditions in fields surrounded by species‐rich hedgerows, where little or no herbicide was used due to the short picking intervals of the crop (P.E. Simmons and Son, personal communication 1st November 2017). This meant that there was a high abundance and species richness of wild flowers within and around the crop.

### 2.3 Quantifying nectar and pollen resources in courgette flowers (2017)

The standing crop of nectar and pollen and the 24‐hr secretion rate of nectar (Corbet, 2003) were quantified to show the availability of pollen and nectar over time as well as to parameterize *Bumble*‐BEEHAVE (Figure 1). The weight of sugar (mg; nectar) and pollen (mg) were calculated per flower. Detailed information about pollen and nectar measurements are in Supporting Information Appendix S1.

### 2.4 Bee visitation to courgette and wild flowers (2016 and 2017)

To quantify *B. terrestris* abundance at courgette flowers, and therefore, their potential pollination efficiency (Figure 1), four 50 m transects were established within the crop from the edge of the crop to the center, 25 m apart. Transects were walked at a steady pace (~5 min each) with observations made 1 m either side and in front of the recorder. This was done three times during the blooming period for each site in 2016 and 2017, resulting in a total of 228 transects surveyed over the 2 years. Sampling was conducted between 08:00 and 10:00 hours (when flowers were open) on sunny to partly cloudy days.

In 2017, additional transects in the crop and the field margins were simultaneously surveyed by two observers from 08:15 to 15:30 hours at ten sites, resulting in an additional 640 transects. This was to capture pollinator activity in the 4 hr either side of courgette senescence, which occurs around 12:00 hours.

All bee species and the plant species they were feeding on, for nectar or pollen, were recorded to species level. However, *B. terrestris *and bees belonging to the *Bombus lucorum *L. complex were all recorded as “*B. terrestris*” due to difficulties in reliably distinguishing workers in the field (Murray, Fitzpatrick, Brown, & Paxton, 2008). Since colonies of *B. terrestris* were added to all fields in 2017, foragers from these colonies are highly likely to have been recorded on pollinator transects.

### 2.5 Pollination of courgette flowers by *B. terrestris* (2017)

#### 2.5.1 Swabbing *B. terrestris* for pollen grains

To quantify the number of courgette pollen grains carried on *B. terrestris*, and therefore, their potential pollination efficiency (Figure 1), *B. terrestris* (*n* = 17) and *A. mellifera* (*n* = 4) were randomly collected from courgette flowers and placed in individual sample pots. Bees were gently cooled under ice packs, and their entire body swabbed with small cubes of glycerin jelly (with fuchsin dye) positioned on the end of cocktail sticks, before they were released. In the laboratory, microscope slides were prepared by melting the piece of glycerin jelly under a coverslip. The number of courgette pollen grains were then counted under a 20× magnification (Kremen, Williams, & Thorp, 2002).

#### 2.5.2 Pollen grains on stigmas

To quantify courgette pollination, pollen accumulation per stigma was quantified (Figure 1). A total of 20 stigmas were removed from pistillate flowers and placed into centrifuge tubes every 90 min from 05:30 to 12:00 hours over 2 days at two different sites (10 stigmas per time point per day). In the laboratory, 1/6 of the stigma (one half of a lobe) was dissected and gently squashed onto a microscope slide; fuchsin jelly was then melted over the stigma, under a coverslip (Kremen et al., 2002). The number of courgette pollen grains were then counted with a 20× magnification and multiplied by six to achieve an estimate of pollen deposition for the whole stigma.

#### 2.5.3 Yield

To further quantify courgette pollination (Figure 1), yield measurements were also taken. To do this, commercial colonies of *B. terrestris* were closed at one field site to quantify courgette yield without managed *B. terrestris *present. This was done over five nonconsecutive days for a total of 100 pistillate flowers (20 flowers per day), following the methodology for “open pollination” in Knapp and Osborne (2017).

### 2.6 Effect of courgette on *B. terrestris* colony development (2017)

#### 2.6.1 Pollen loads from *B. terrestris*

To quantify the proportion of courgette pollen in *B. terrestris*’ diet (Figure 1), “forager trap modules” (Martin et al., 2006) were placed onto all commercial colonies within a field for around 45 min, between 07:00 and 09:00 hours. Once trapped on returning from a foraging trip, workers were narcotised in situ using CO_2_ for 30 s and the number of bees carrying (and not carrying) pollen loads were recorded. One pollen pellet from one of the corbiculae on each bee, that is, half of their total pollen load, was placed into a centrifuge tube and taken back to the laboratory. Here, all pollen loads (*n* = 394) were sorted to color and all yellow pollen loads checked to see if they were courgette, which has large (180–200 µm in diameter) and distinctive pollen grains (Nepi & Pacini, 1993). A subset (*n* = 56) of all pollen loads were identified to species where possible using Sawyer (1981) and a microscope. All foragers were returned to their colony within an hour of being caught. Pollen loads were taken from 42 colonies across the 14 sites and each site was surveyed on a separate day.

#### 2.6.2 Habitat maps

Habitat maps for each study site were required to estimate the amount of forage and nesting sites, that is, seminatural habitat and mass‐flowering crops, available to bumblebees in the landscape (Figure 1) (Kremen, Williams, Bugg, Fay, & Thorp, 2004; Westphal et al., 2003). To create these maps, seminatural habitat (woodlands and heathland), improved grassland, and mass‐flowering crops (courgette and maize) were recorded in 750 m radii of each field site in 2017 (*n* = 14). This was done by ground truthing satellite imagery and adapting Land Cover 2007 data (Centre for Ecology & Hydrology, 2011) using ArcGIS 10.2.2. Each site had varying quantities of crop and habitat types (Supporting Information Figure S1).

#### 2.6.3 *Bumble*‐BEEHAVE simulations using BEE‐STEWARD

Simulations were run in BEE‐STEWARD (www.beesteward.co.uk), a software tool that combines in a user‐friendly way the bumblebee model *Bumble*‐BEEHAVE and the landscape defining features of BEESCOUT (Becher et al., 2016). BEESCOUT was developed as the landscape module for the honeybee model BEEHAVE (Becher et al., 2014) and for *Bumble‐BEEHAVE *(Becher et al., 2018), and creates input files from images of landscape maps. These input files define the number and specification of food sources such as, nectar and pollen, flowering phenology, and therefore, represent landscapes in the BEEHAVE and *Bumble*‐BEEHAVE models. BEE‐STEWARDS’ interface also enables users to simulate the effects that different management options, such as changing crop types will have on bumblebee population dynamics.

The default settings for *Bumble*‐BEEHAVE start simulations at the beginning of the year with 500 *B. terrestris* queens who randomly emerge from hibernation around 1st of April (±28 days *SD*), following a normal distribution. In the model, queens can nest in all types of seminatural habitat implemented in the model: heathland, species‐rich grassland, hedgerow, scrub and woodland (Becher et al., 2018). The number of nests in the landscape is a result of the number of queens emerging from hibernation and their daily probabilities to find a suitable nest site or to die (Becher et al., 2018).

Habitat types are defined by the presence and abundance of 44 forage plants which provide nectar and or pollen during specified flowering periods. Once a simulated queen has found suitable nesting habitat, she must collect sufficient pollen and nectar resources before laying her first batch of eggs. She will then continue to split her time between foraging and brood care until the first adult workers emerge. The queen will then focus on egg‐laying while workers divide their time between brood care and foraging. Foraging choices are based on maximizing foraging rate (pollen) or energetic efficiency (nectar), which depends on distance, handling time, and the degree of patch depletion. The probability of a bee detecting a new patch is based on the distance of the food source from a colony. Toward the end of colony development female larvae may develop into queens, and the original queen switches from laying diploid eggs to haploid, male eggs. Once new queens are developed they leave their colony, mate, and hibernate prior to emergence the following year. For a detailed model description, see Supplementary material S03 (“ODD protocol”) of Becher et al. (2018).

BEE‐STEWARD's flexible input settings meant that habitat types recorded on surveys, which were not already in the mode, that is, courgette, heathland, and improved grassland could be easily parametrized in the input files for analysis (Supporting Information Table S1). Courgette fields were specified as either “early courgette,” flowering from the beginning of June until the middle of July, or “late courgette” flowering from the middle of July until the end of August, to reflect the cropping practices of courgette production in the UK. A map of each study site was separately input into the model and manually edited (if needed) using the functions available within the program (Becher et al., 2016).

In order to reduce computational time and to ensure that simulations were based solely on populations in equilibrium (Hui, 2006), a set of preliminary simulations were run in landscapes with no courgette, where courgette fields had been temporarily removed, as a baseline. To determine a suitable number of initial queens for all landscapes, simulations were started with 500 hibernating queens and run over 15 years in each landscape 20 times. The number of queens was then plotted over time to see at what number of queens the population appeared to reach equilibrium (Supporting Information Figure S2). This resulted in 500 hibernating queens as a conservative estimate for all landscapes and simulations. To determine the length of simulations (i.e., time taken to reach equilibrium), simulations were run starting with a population size that was either close to the estimated number of hibernating queens (500) or above it (1,000) across all landscapes (with no courgette) 20times, over 20 years. The population was assumed to be in equilibrium, once both growth curves had converged (Supporting Information Figure S3). Year 11 was taken as the year where all landscapes were in equilibrium.

The effect of courgette on *B. terrestris* population dynamics was explored by reclassifying courgette fields in landscape maps of actual study sites to either “early season courgette,” “late season courgette” or “no courgette” in BEE‐STEWARD. This created three different cropping scenarios for simulations in *Bumble*‐BEEHAVE: (a) no mass‐flowering crop (baseline), (b) early season courgette, and (c) late season courgette (Supporting Information Table S1). All simulations were run 10 times per landscape and cropping scenario, totaling 420 simulations.

The average number of overwintering queens, colonies, and adult workers were calculated daily for each landscape over 11 years.

### 2.7 Statistical analysis

All analyses were carried out using R (R Core Team, 2017). For empirical data, independent sample *t* tests were used to compare the differences in mean sugar production (g) between staminate and pistillate flowers (over 24 hr and every 90 min), pollen depletion (mg/flower) between 05:30 and 10:00 hours, pollen accumulation on stigmas (grains/stigma) between 05:30 and 11:30 hours, and *B. terrestris* abundance in the margin and cropped area per hour.

For simulated data, the effect of cropping scenario (fixed effect) was explored in relation to the peak number of hibernating queens (day 365), adult workers (day 149), and colonies (day 149) in year 11 using linear mixed‐effects models with site specified as a random effect. Post hoc Tukey tests were calculated using the multcomp package (Hothorn, Bretz, & Westfall, 2008). All means are presented with their associated standard error unless otherwise stated.

## 3 RESULTS

### 3.1 Nectar and pollen measurements from courgette

**Figure 2:**
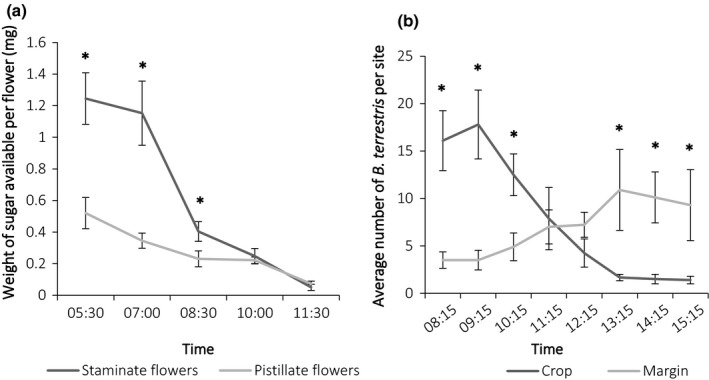
(a) Weight of sugar available (±*SE*) every 90 min for 50 staminate and 50 pistillate flowers (500 flowers in total) and (b) average number of *Bombus terrestris* in the crop and on the margin over time, data were summed per transect in either the crop or on the margin and averaged by site (*n* = 10), all sites contained commercial colonies of *B. terrestris*. Significant independent *t* tests are indicated with an asterisk (*) for each time point (*p* < 0.05)

The secretion rate of nectar, that is, the weight of sugar produced over 24 hr from bagged flowers was greater (although not statistically, *T*_78_ = −1.94, *p* = 0.06) for pistillate flowers (34.41 ± 2.67 mg per flower, *n* = 40) than staminate flowers (26.59 ± 1.56 mg per flower, *n* = 40). These estimates were much higher than the standing crop of nectar, that is, weight of sugar available at a given time point per flower, which at 05:30 hours was just 0.52 ± 0.09 mg for pistillate flowers (*n* = 50) and 1.24 ± 0.16 mg for staminate flowers (*n* = 50; Figure 2a). By 11:30 hours, nearly all sugar was depleted from both staminate (0.05 ± 0.01 mg, *n* = 50) and pistillate (0.07 ± 0.01 mg, *n* = 50) flowers (Figure 2a).

The weight of pollen produced over 24 hr from bagged flowers was 18.04 ± 0.84 mg per staminate flower (*n* = 40). Again, this was much greater than the weight of pollen available from unbagged flowers, which was estimated to be 10.96 ± 1.39 mg per flower at 05:30 hours (*n* = 20). From 05:30 to 10:00 hours, there was no significant loss (*T*_37_ = −1.22, *p* = 0.23) of pollen (10:00 hours =8.37 ± 1.64 mg) suggesting that much of the pollen is removed around anthesis when the very first pollinator visits occur.

### 3.2 Visitation to courgette and wildflowers

**Figure 3:**
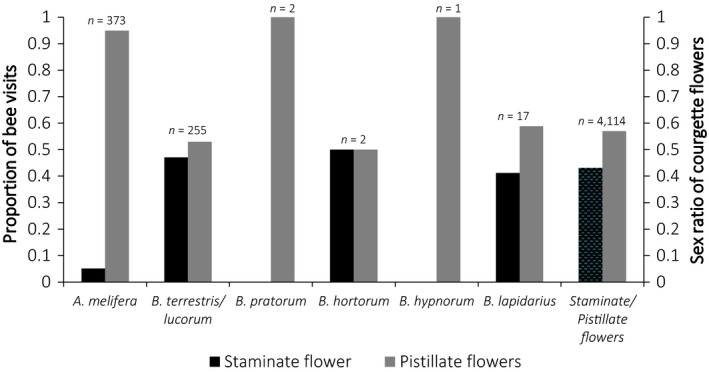
Proportion of nectar visits to staminate and pistillate flowers for *Apis mellifera*, *Bombus terrestris*/*lucorum*, *Bombus pratorum*, *Bombus hypnorum,* and *Bombus lapidarius* recorded on pollinator transects in 2016 and 2017, as well as the proportion of staminate and pistillate flowers on floral transects in 2016 and 2017. Data were pooled from all transects conducted in the cropped area of 19 fields

*Apis mellifera* and *B. terrestris* were the most abundant pollinator species observed visiting courgette flowers across the 2 years of this study, although commercial colonies of *B. terrestris* were added to fields in 2017 (Figure 3). *Bombus terrestris* showed a more equal preference to staminate and pistillate flowers then *A. mellifera* (Figure 3).

In the morning*, B. terrestris* was significantly more abundant in the crop when courgette flowers were open and providing nectar, than in the margin (Figure 2b). However, in the afternoon *B. terrestris* were significantly more abundant in the margin than in the crop when courgette flowers are closed and no longer providing nectar (Figure 2b).

### 3.3 Pollination of courgette flowers

*Bombus terrestris* carried an average of 1,866 ± 476 (*n* = 13) pollen grains on their bodies, more than *A. mellifera* which carried an average of 122 ± 39 (*n* = 4) pollen grains on their bodies.

By 11:30 hours, an average of 4,749 ± 441 (*n* = 18) pollen grains had been deposited onto each stigma, significantly more (*T*_32_ = −5.52, *p* = <0.001) than at 05:30 hours 1,879 ± 276 (*n* = 16).

The percentage of open‐pollinated pistillate flowers setting fruit was very high across the 5 days of surveying at 97% ± 2% (*n* = 96).

### 3.4 Effect of courgette on *B. terrestris* colony development

#### 3.4.1 Pollen loads

None of the 394 pollen loads collected from the 42 colonies of *B. terrestris* contained courgette pollen (Supporting Information Table S2). Brassica spp. (15), bramble (11), and common poppy (seven) were the most common pollen species identified out of a subsample (*n* = 56) of pollen loads (Supporting Information Table S2). Consequently, all courgette flowers were specified as having a pollen resource value of zero in BEE‐STEWARD (Supporting Information Table S1).

#### 3.4.2 Bumble‐BEEHAVE simulations using BEE‐STEWARD

**Figure 4:**
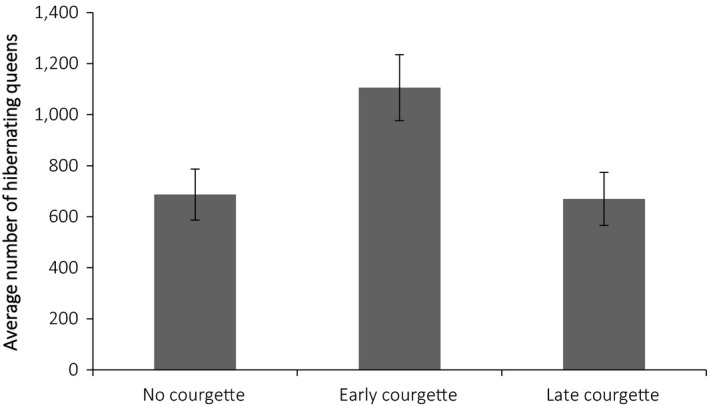
Average number of hibernating queens ± *SE* on the last day of year (year 11) for each cropping scenario. Data were averaged across the 10 repeated runs and 14 study sites

**Figure 5:**
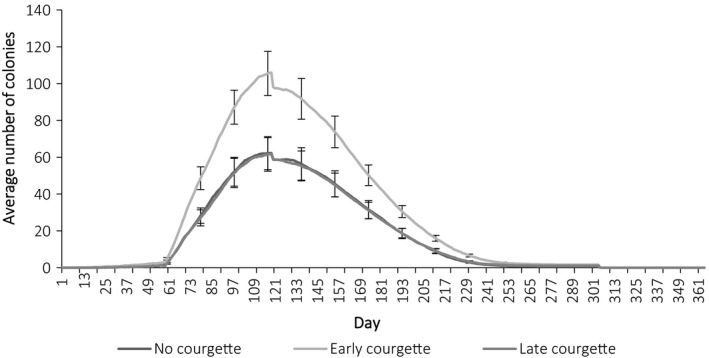
Average number of colonies (±*SE* every 20 days) over the course of a year (year 11) for each cropping scenario. Data were averaged across the 10 repeated runs and 14 study sites

**Figure 6:**
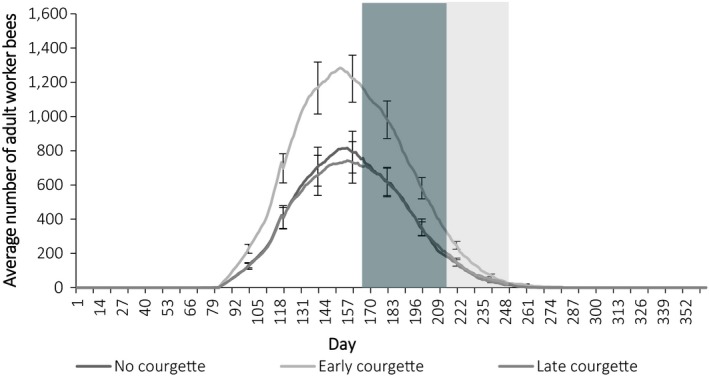
Average number of adult worker bees (±*SE* every 20 days) over the course of a year (year 11) for each cropping scenario. Shaded areas show the flowering times of courgette, early courgette is shown in dark gray, late courgette shown in light gray. Data were averaged across the 10 repeated runs and 14 study sites

Landscapes with early courgette had a higher “carrying capacity” for queen bumblebees, determined by the number of overwintering queens on the last day of the year compared to those with no courgette (contrast estimate −424.66 ± 26.92 *Z = *15.77, *p* = <0.001) and late courgette (contrast estimate −436.89 ± 26.29, *Z* = 16.64, *p* = <0.001; Figure 4). Likewise, early courgette resulted in the establishment of more colonies in the landscape compared to no courgette (contrast estimate −30.62 ± 1.96, *Z = *−15.65, *p* = <0.001), and late courgette (contrast estimate −31.44 ± 1.91, *Z = −*16.44, *p* = <0.001) on day 149 (Figure 5). This resulted in more adult workers during peak foraging activity (day 149) across early courgette landscapes compared to no courgette (contrast estimate −481.37 ± 37.5, *Z = *−14.59, *p* = <0.001) and late courgette (contrast estimate −534.88 ± 36.66, *Z = *−14.59, *p* = <0.001) landscapes (Figure 6). Indeed, the year on year effect of early courgette also increased the abundance of foragers early in the season, before courgette flowering (Figure 6). The phenology of early season courgette (flowering from beginning of June to the middle of July) is more closely related to forager activity, indicated with the baseline no courgette, and longer in duration than late season courgette (flowering from middle of July until the end of August; Figure 6).

## 4 DISCUSSION

This study clearly demonstrates a mutualistic relationship between courgette flowers and *B. terrestris *that is beneficial to both, improving pollination success and colony dynamics (Bailes, Ollerton, Pattrick, & Glover, 2015; Holzschuh et al., 2016). Courgette, offers an abundant source of nectar to attract pollinators to its flowers for pollination (Vidal, Jong, Wien, & Morse, 2006). Indeed per m^2^, courgette offers more nectar (0.35 ml) than oilseed rape (0.30 ml), and field bean (0.092 ml) (Becher et al., 2016), and is therefore a high value mass‐flowering crop in terms of nectar production.

Results showed that over 24 hr pistillate flowers produce significantly more sugar than staminate flowers. This overall higher sugar content combined with nectaries which are harder to access than staminate flowers is thought to be why bee species show a preference for, and spend longer at pistillate flowers (Artz & Nault, 2011; Nepi & Pacini, 1993; Phillips & Gardiner, 2015; Tepedino, 1981). At a field scale, *B. terrestris* also showed a strong fidelity to courgette, visiting crop flowers more often than wildflowers in the hedgerows, in the morning when courgette flowers were open, providing the first empirical evidence of *B. terrestris* fidelity to a *Cucurbita* crop (Petersen, Reiners, & Nault, 2013).

In this study, the majority of courgette pollen was removed around anthesis during the very first pollinator visits (Phillips & Gardiner, 2015; Stanghellini, Schultheis, & Ambrose, 2002). However, personal observations showed *B. terrestris* removing excess courgette pollen grains from their bodies early in the morning, supporting the findings of Nepi and Pacini (1993). Nonetheless, *B. terrestris* was still observed to carry more loose pollen grains on their body, and therefore, have a higher pollination potential than *A. mellifera*. Indeed pollen was still transferred to stigmas well after anthesis and by the end of the morning, stigmas had received an adequate number of pollen grains (4,749 ± 441) for optimum fruit set as ~1,200 are thought to be required for maximal fruit set in pumpkin (Vidal, Jong, Wien, Morse, & a., 2010). This was evidenced by the high percentage fruit set, and therefore, very low pollination deficit in this study. Despite courgette pollen being relatively high in protein (Petersen et al., 2013), its large sticky grains may make it difficult for *B. terrestris* to collect (Vaissière & Vinson 1994). *Bombus terrestris* may also avoid collecting *Cucurbita *pollen, since as a generalist species it can visit alternative, more easily obtainable pollen, unlike *Peponapis* and *Xenoglossa* spp. which as *Cucurbita* specialists are thought to rear their offspring exclusively on *Cucurbita* pollen (Tepedino, 1981). This may be why no pollen loads from returning *B. terrestris* foragers contained courgette pollen.

After courgette flower senescence (within a day) *B. terrestris* appeared to “switch” from courgette to hedgerow flowers, evidenced by the diverse range of pollen loads collected from returning *B. terrestris* foragers. While some of these plant species may occur in hedgerows immediately surrounding courgette fields, others may be from species located further away. This highlights the importance of maintaining wildflowers at different spatial scales to fulfill bees’ requirements for nectar and pollen beyond that of the focal crop. Indeed flower‐rich areas have been shown to increase colony density (Wood et al., 2015) and food supplementation shown to increase colony development, particularly of queen and male bumblebees (Pelletier & McNeil, 2003). However, the extent to which pollinators are attracted into mass‐flowering crops will vary depending on the relative quality and quantity of floral resources in the mass‐flowering crop and nearby seminatural habitat. In this study, it appears that wildflowers near to mass‐flowering courgette facilitate pollination services to courgette, supporting bumblebee nutrition without distracting bees from courgette flowers. Indeed, wildflower species richness in courgette fields has been shown to be the most important factor for determining bumblebee abundance at courgette flowers (Knapp, Shaw, & Osborne, 2018). Therefore, wildflowers around courgette fields could attract bumblebees to courgette flowers also provide additional forage.

Given courgette's bountiful, yet transient supply of nectar, bumblebee population dynamics were shown (using *Bumble*‐BEEHAVE) to improve in landscapes with early flowering courgette compared to a no courgette baseline. As bumblebee foragers are generally most active mid‐summer, early courgette was the best cropping scenario for concurrently achieving more forager visits (pollination potential) and more food (nectar only) to be brought back to the colony. However, bees can only benefit from the additional energy provided by courgette nectar, which will help to reduce foraging efforts, if protein‐providing pollen is also available to raise their brood. Empirical data showed that, within a day, bees were able to utilize courgette and wildflowers for nectar (Figure 2b) as well as wildflowers for pollen (Supporting Information Table S2). This supports model results which showed at a coarser temporal scale that with more nectar, colonies were able to grow and subsequently forage on more, additional resources for pollen. Subsequently, early courgette supports more adult workers (foragers), colonies, and hibernating queens for subsequent years compared to late, and no courgette landscapes. Thus, planting early courgette and late courgette in fields adjacent to each other could improve forager numbers in late courgette and further improve bumblebee populations for subsequent years (Riedinger, Renner, Rundlöf, Steffan‐Dewenter, & Holzschuh, 2014).

The phenological matching of crops with key periods of pollinator activity is thought to be why the presence of oilseed rape in the landscape (early in the season) can improve the reproductive potential of *Osmia bicornis *L. (Holzschuh, Dormann, Tscharntke, & Steffan‐Dewenter, 2013; Jauker, Peter, Wolters, & Diekötter, 2012), but not *Bombus pascuorum* S. (Herrmann, Westphal, Moritz, & Steffan‐Dewenter, 2007) and *B. terrestris* (Westphal et al., 2009). This is because while oilseed rape can improve colony establishment and growth of bumblebees, the lack of resources later in the season mean there is no increase in the number of males or queens produced (Herrmann et al., 2007; Westphal et al., 2009). This lack of phenological matching is also true of late courgette which despite offering resources later in the season (unlike oilseed rape) still misses the key period of bumblebee foraging. However, Rundlöf, Persson, Smith, and Bommarco (2014) observed more queen and male bumblebees on transects around fields of late‐flowering red clover, suggesting results could be specific to flower and pollinator species. Interestingly, the average number of colonies per landscape decline around day 119, which may be a result of willow species, common to hedgerows and scrub in *Bumble*‐BEEHAVE's input files, no longer flowering.

## 5 CONCLUSION

Combining empirical data on pollinator visitation, nectar and pollen availability, and pollination efficiency, with model simulations have provided a unique insight into the mutualistic relationship between *B. terrestris *and the mass‐flowering crop, courgette. Flower‐scale data (within a day) showed how effective a pollinator *B. terrestris* is in courgette and the extent to which they utilize courgette flowers for pollen and nectar. Based on this information, *Bumble*‐BEEHAVE was parameterised to show the effect of courgette management at a crop‐scale (within a year) which, while theoretical, is consistent with empirical knowledge.

Broadly, these findings show that matching crop phenology with key periods of forager activity can be an effective way of improving bumblebee population dynamics and pollination efficiency. Increased understanding of a plant‐pollinator mutualism at different temporal and spatial scales means that management recommendations can be made. For growers, this may mean planting mass‐flowering crops with complementary phenologies, such as early and late courgette, in fields adjacent to each other. For conservationists, it may mean recognizing the importance of courgette, alongside other mass‐flowering crops, as valuable forage resources for bumblebees, while continuing to promote additional sources of forage to fulfill bees’ nutritional requirements over space and time. In doing so, it could be possible to simultaneously improve pollination services and bumblebee populations in intensive farmland.

## AUTHORS’ CONTRIBUTIONS

JK and JO conceived the ideas and designed methodology; JK and CR collected the data; JK and MB analyzed the data; MB and GTD created the BEE‐STEWARD software and provided support on simulations. JK led the writing of the manuscript. All authors contributed critically to the drafts and gave final approval for publication.

## DATA ACCESSIBILITY

The research data supporting this publication are openly available from the University of Exeter's institutional repository at: https://doi.org/10.24378/exe.823.
