# Supplementary material for: Bombus terrestris in a mass‐flowering pollinator‐dependent crop: A mutualistic relationship?
Source: Ecol Evol. 2018 Dec 18;9(1):609–18. doi: 10.1002/ece3.4784 (PMC6342091; doi:10.1002/ece3.4784)
Supplement: Supplementary file 1 [file ECE3-9-609-s001.docx]

**Figures S1 - S3**

Fig. S1 Area (m²) ± *SD* of the different habitat types providing pollen and/or nectar across the 14 landscapes. In model simulations the area of courgette was specified as either ‘early courgette’ or ‘late courgette’ (see Table A 4.1), or ‘no courgette’ (no habitat specified in the model). *Simulated B. terrestris* were able to nest in heath, hedgerow, meadow, scrub, and woodland.

Fig. S2 Average number of hibernating queens ± *SD* (shaded) across 14 landscapes with no courgette present (baseline) over 15 years. Data were simulated 20 times for each landscape.

Fig. S3 Average numbers of hibernating queens simulated for the study site 10, the last of the 14 different landscapes to reach equilibrium, i.e. when both growth curves have converged; taken as year 11 (a conservative estimate). Data were simulated 20 times for each landscape.
