# Supplementary material for: Bombus terrestris in a mass‐flowering pollinator‐dependent crop: A mutualistic relationship?
Source: Ecol Evol. 2018 Dec 18;9(1):609–18. doi: 10.1002/ece3.4784 (PMC6342091; doi:10.1002/ece3.4784)
Supplement: Supplementary file 2 [file ECE3-9-609-s002.docx]

**Table S1 Input parameters for additional crop and habitat types for BEE-STEWARD.**

| Crop type | Flower species | Flower density (flowers/m²) | Pollen (g/ flower) | Nectar (ml/ flower) | Sugar concentration of nectar (mol/l) | Flowering start day | Flowering stop day | Corolla depth (mm) | Nectar flower volume (myl/ flower) | Internal flower handling time (seconds) | Notes |
| --- | --- | --- | --- | --- | --- | --- | --- | --- | --- | --- | --- |
| Courgette | Early courgette | 3 | 0* | 0.0765 | 1.4660 | 163 | 212 | 0 | 76.47 | 2.5 | 1) |
|  | Late courgette | 3 | 0* | 0.0765 | 1.4660 | 213 | 251 | 0 | 76.47 | 2.5 |  |
| Improved grassland | Dandelion | 0.05 | 0.0004 | 0.0005 | 1.2947 | 1 | 364 | 1.2 | 0.4702 | 0.6 | 2) |
|  | White clover | 2.34 | 0.0004 | 0.0007 | 0.9803 | 151 | 272 | 2 | 0.6666 | 0.6 |  |
| Heath | Ling | 465 | 0** | 0.0001 | 1.17 | 182 | 273 | 4 | 0.0787 | 0.6 | 3) |
|  | Bell Heather | 7.17 | 0** | 0.0002 | 1.17 | 121 | 334 | 5.5 | 0.1997 | 0.6 |  |
|  | Cross-leaved Heather | 2.14 | 0** | 0.0002 | 1.17 | 152 | 273 | 6.5 | 0.1742 | 0.6 |  |
|  | Bilberry | 9.23 | 0** | 0.0026 | 1.17 | 91 | 181 | 5 | 2.6215 | 0.6 |  |

**Notes (Table S1):**

1) Empirical observations. * Pollen values set to 0 as no bees were observed bringing back courgette pollen to their colonies.

2) Nectar and pollen were already in the model; however, flower species and flower density were based on empirical observations (Twiston-Davies et al. 2018, unpublished data). Flower species and density were estimated from 10, 1m^2^ quadrats across 4 fields of improved grassland (40 quadrats overall).

3) Data from Baude *et al*. (2016). ** No pollen data available for these species.
