# Supplementary material for: Bombus terrestris in a mass‐flowering pollinator‐dependent crop: A mutualistic relationship?
Source: Ecol Evol. 2018 Dec 18;9(1):609–18. doi: 10.1002/ece3.4784 (PMC6342091; doi:10.1002/ece3.4784)
Supplement: Supplementary file 3 [file ECE3-9-609-s003.docx]

**Table S2 Plant species identified from a subsample of pollen loads n=56, from the total 394 loads collected from 42 colonies at 14 sites. None of the yellow pollen was courgette pollen.**

| Species name | Common name | Number of pollen loads |
| --- | --- | --- |
| *Brassica spp.* | Brassica spp. | 15 |
| *Rubus fruticosus* | Bramble | 11 |
| *Papaver rhoeas* | Common poppy | 7 |
| *Veronica filiformis* | Speedwell | 4 |
| *Helianthemum chamaecistus* | Common rockrose | 3 |
| *Linaria vulgaris* | Common toadflax | 3 |
| *Verbascum thapsus* | Great mullein | 3 |
| *Echium vulgare* | Viper’s bugloss | 2 |
| *Hedera helix* | Common ivy | 2 |
| *Ribes sanguineum* | Flowering currant | 2 |
| *Calystegia sepium* | Hedge bindweed | 1 |
| *Centaurea cyanus* | Cornflower | 1 |
| *Centranthus ruber* | Red valerian | 1 |
| *Heracleum sphondylium* | Hogweed | 1 |
