# Supplementary material for: Bombus terrestris in a mass‐flowering pollinator‐dependent crop: A mutualistic relationship?
Source: Ecol Evol. 2018 Dec 18;9(1):609–18. doi: 10.1002/ece3.4784 (PMC6342091; doi:10.1002/ece3.4784)
Supplement: Supplementary file 4 [file ECE3-9-609-s004.docx]

**Appendix S1 Nectar and pollen quantification methods**

For each flower, full access to the nectary was achieved by removing the stigma or stamen with a scalpel. The standing crop of nectar was calculated from 50 staminate and 50 pistillate flowers every 90 minutes from 05:30 to 12:00 h over five days (10 staminate and 10 pistillate flowers per time point per day). This volume is likely less than when the same flower is repeatedly surveyed due to the flower replenishing resources (Corbet 2003). In addition, the 24 hour secretion rate of nectar was calculated by securing PVC mesh bags to flowers with wire ties the day before expected anthesis. Bags had a mesh size of 0.2mm, designed to be permeable to wind and rain yet exclude any pollinators (Corbet 2003). Bags were then removed around 11:00 h and all nectar extracted individually from 40 staminate and 40 pistillate flowers, over four days (10 staminate and 10 pistillate flowers per day).

Nectar volume (μl) was measured using glass microcapillary tubes (sizes 2, 5, 10 and 20 μl microcaps, Drummond Scientific, Broomall PA, USA) and nectar sugar concentration (mg/mg) was measured using a hand-held refractometer modified for small volumes (Eclipse, Bellingham & Stanley, Tunbridge Wells, UK). Nectar sugar concentration as measured by the refractometer, i.e. weight of solute per weight of solution (*C*; mg/mg), was converted to nectar sugar concentration in terms of weight of solute per volume of solution (*d*; mg/μl) using *d* = (0.0037291*C* + 0.0000178*C*^2^ + 0.9988603) (from Prŷs-Jones & Corbet, 1991). The weight of sugar produced per flower over 24 h (*w*) (mg) was then calculated using *w* = *dvc,* where *v* is volume of nectar (μl) and *c* is sugar concentration of nectar as a proportion (mg/μl).

The amount of pollen (mg per flower) available at a given time point was quantified from 20 stamens which were removed from staminate flowers every 90 minutes from 05:30 to 12:00 h over two days (10 stamens per time point per day) to show pollen depletion within a day. In addition, the total amount of pollen produced in 24 hours was quantified from 40 stamens which were removed from staminate flowers (secured with PVC mesh bags the day before expected anthesis) over two days (20 stamens per day). All stamens were placed in centrifuge tubes in the field.

Owing to the vast quantities of pollen on stamens, pollen was weighed rather than counted. This was done by adding 1ml of distilled water, using a pipette, to centrifuge tubes containing stamens, agitating them for 10 seconds at 12,000 rpm, removing stamens with forceps, and centrifuging at 12,000 rpm for 10 minutes. The supernatant was then removed using a pipette and the centrifuge tube placed in a drying cabinet at approximately 40 °C for 24 hours. The samples and centrifuge tubes were weighed on a balance before being washed and returned to the drying cabinet for a further 24 hours and weighed again. The weight of the empty centrifuge tube was then subtracted from the centrifuge tube containing the pollen to provide the weight of pollen per flower (mg per flower).
